# Dairy Caretaker Perspectives on Performing Euthanasia as an Essential Component of Their Job

**DOI:** 10.3390/ani11020289

**Published:** 2021-01-24

**Authors:** Ivette Noami Román-Muñiz, Mary Caitlin Cramer, Lily N. Edwards-Callaway, Lorann Stallones, Elizabeth Kim, Sofia Thompson, Hailey Simpson, Sage Mijares

**Affiliations:** 1 Department of Animal Sciences, College of Agricultural Sciences, Colorado State University, Fort Collins, CO 80523, USA; catie.cramer@colostate.edu (M.C.C.); lily.edwards-callaway@colostate.edu (L.N.E.-C.); sofia464464@gmail.com (S.T.); hailey.simpson@colostate.edu (H.S.); 2 Department of Psychology, Colorado State University, Fort Collins, CO 80523, USA; lorann.stallones@colostate.edu (L.S.); elizabeth.kim@colostate.edu (E.K.); 3 College of Veterinary and Biomedical Sciences, Colorado State University, Fort Collins, CO 80523, USA; mijaress@rams.colostate.edu

**Keywords:** on-farm euthanasia, dairy worker wellbeing, dairy caretaker perceptions, euthanasia-related stress, dairy animal wellbeing, human–animal bond

## Abstract

**Simple Summary:**

Although on-farm euthanasia is a critical component of dairy management and provides a way to alleviate animal suffering, little is known about dairy caretaker perspectives on their role in euthanasia, their comfort level with the procedures, and the impact of practicing euthanasia on their wellbeing and mental health. Thematic analysis of focus groups and interviews of Colorado dairy caretakers revealed a human–animal bond and empathy toward animals in all participants. Training practices were varied and correlated with knowledge about euthanasia procedures and language used to discuss euthanasia. Participants reported that little communication about the stress related to performing euthanasia occurred on the farm, and there was no awareness of mental health resources available to caretakers. Testing of interventions that can support animal caretakers by reducing euthanasia-related stress and improving emotional wellbeing, and efforts to increase awareness of mental health recourses are clear needs for dairy operations.

**Abstract:**

The objectives of this study were to identify caretakers’ perceptions and stressors related to performing on-farm euthanasia as well as potential ideas for intervention strategies to alleviate negative effects of performing euthanasia on caretaker well-being. Additionally, we aimed to determine how euthanasia methods, procedures, and training correlate with dairy caretakers’ attitudes toward performing on-farm euthanasia and their job satisfaction and sense of well-being. Thirty-eight dairy caretakers (19 workers, 15 supervisors, 3 owners, 1 veterinarian) participated in focus groups or interviews conducted and recorded on five Northern Colorado dairies. Thematic analysis of focus group and interview transcripts revealed seven recurring themes. Variation in available training and euthanasia protocols among dairy farms was evident. There was a lack of awareness regarding available mental health resources and little communication between farm personnel about euthanasia-related stress. Training was correlated with caretaker knowledge about euthanasia procedures and the language used to refer to euthanasia. Human-animal bonds and empathy toward animals were evident in participants regardless of training, job position, or dairy experience. Interventions such as training programs, mental health resources, and other support systems should be tested for effectiveness in addressing euthanasia-related stressors, improving euthanasia decision-making and practice, and increasing animal wellbeing on dairy farms.

## 1. Introduction

Euthanasia is an essential management tool in all animal industries and is used as a means to end animal suffering when recovery is unlikely, and quality of life is compromised. Despite the fact that animal caretakers recognize euthanasia as a mechanism to alleviate pain and suffering, deciding when to euthanize, performing euthanasia, and witnessing euthanasia are difficult duties for those who care for the animals daily. Evidence of the “moral stress” [1] caused by performing euthanasia has been well documented in animal shelters and at veterinary clinics. It is known that employees who perform euthanasia as a part of their job experience work-related stress, stress-induced physical ailments, depression, unresolved grief reactions, work-family conflict, burnout, and dissatisfaction with their work [2,3,4,5]. Animal shelter staff report sadness, crying, anger, and depression in response to performing repeated euthanasia [4]. Performing euthanasia has been indicated as contributing to the prevalence of suicide risk and psychological distress in veterinarians [6,7].

While considerable research has been conducted exploring the impacts of performing euthanasia on veterinarians and animal shelter workers, research is more limited regarding impacts of performing euthanasia on animal caretakers and veterinarians in the livestock industry. Terminology like the “caring-killing paradox” has been previously used to describe the emotional strain of individuals responsible for euthanizing animals, but limited research has been conducted to identify emotional factors at play regarding impacts of euthanasia on farm workers in the dairy industry [8]. Dairy workers have expressed a desire to alleviate animal suffering by utilizing euthanasia but have also expressed a wide range of emotional states and levels of emotional distress as a result of euthanizing dairy animals [8]. In a review of challenges associated with timely euthanasia on dairy farms, factors related to animal caretaker perceptions and knowledge were described including inconsistent employee training, lack of protocols, consideration for animal quality of life, and impacts of the human-animal bond [9]. Understanding the impact of euthanasia on caretakers is the first step to improving euthanasia-related stress on dairy farms.

Dairy workers experience a variety of factors that place them at risk for developing physical and mental health challenges as a result of their work [10]. Lack of health insurance and language barriers are the most common barriers to receiving medical care [10]. In addition, dairy workers typically work overtime performing physically and mentally demanding jobs, including euthanasia [11]. Dairy workers report a high number of work-related injuries, limited awareness of the risks inherent to dairy work, and a general perception that work-related injuries are not preventable [12]. The psychosocial environment of the dairy also contributes to poor occupational safety and health [13]. Previous studies have established that performing euthanasia places workers at risk for stress-induced physical ailments and depression [2,3,4,5]. Many dairy workers are directly involved with euthanizing animals or making euthanasia decisions. Wagner et al. [8] reported that 84.8% of dairy workers indicated that they were responsible for making euthanasia decisions and 59.2% of dairy workers indicated that they were responsible for performing euthanasia.

Animal shelter workers who regularly perform euthanasia as part of their job cope with euthanasia-related work stress in different ways [3]. Many factors influence coping strategy and coping strategy success [14]. Shelter workers indicate that counseling, job rotation, job assistance, breaks and time off, support groups, meetings, skill-based trainings, coping seminars, and employee appreciation are strategies that help them cope with euthanasia-related stress [15]. Animal shelter euthanasia technicians indicate that “simply acknowledging their emotions” is also a successful way to cope with euthanasia-related stress [16].

Additionally, training dairy workers to be confident in their abilities to make timely decisions about euthanasia is critical to safeguarding human safety on-farm [8]. While caretaker training and appropriate euthanasia methods may improve physical human safety, neither aims to assist workers with coping with the mental stress caused by performing euthanasia. Understanding the perceptions of dairy workers specifically related to training and support resources for euthanasia is critical for maintaining and improving both worker and animal well-being on-farm.

The objectives of this pilot study were to identify caretakers’ perceptions and stressors related to performing on-farm euthanasia as well as potential ideas for intervention strategies to help alleviate negative effects of performing euthanasia on caretaker well-being. Additionally, we aimed to determine how euthanasia methods, procedures, and training affect dairy caretakers’ attitudes toward performing on-farm euthanasia and their job satisfaction and sense of well-being. We hypothesize that euthanasia training and management vary among dairy operations and are correlated with caretaker knowledge, attitudes and perceptions regarding euthanasia.

## 2. Materials and Methods

### 2.1. Study Population and Recruitment

Because of the topic being explored, selection of dairy farms to be recruited was not randomized. Instead, dairy operations with a long history of collaboration with Colorado State University’s Department of Animal Sciences were contacted by electronic mail or phone call. Permission was obtained to visit them and recruit animal caretakers to participate in focus groups or interviews. Workers, supervisors, owners, and veterinarians from the targeted dairies were all eligible to participate and were collectively considered dairy caretakers. Caretakers that agreed to participate in the focus groups were offered a $25 gift card for their time.

### 2.2. Data Collection

Focus groups and interviews were conducted on each participating dairy at a convenient date and time that did not interfere with work shifts during the summer of 2019. On each participating dairy, focus groups and interviews took place in a designated area, away from other personnel to maintain confidentiality and allow for honest answers and participation of all caretakers in the group. Refreshments were provided to create a comfortable, welcoming environment conducive to sharing ideas. Participants were identified by their job category (workers, supervisors, owners, veterinarians) and interviewed in their preferred language (English or Spanish). Focus groups with workers were conducted separately from participants in all other job categories. Within some of the job categories there was only one individual, so interviews were conducted in lieu of focus groups. Two members of the research team (one of them bilingual) and one graduate student facilitated and audio-recorded the focus groups and interviews. Twenty questions were asked of the participants (Appendix A), with relevant follow-up questions asked when appropriate.

### 2.3. Data Analysis

All recordings were transcribed and translated into English by one bilingual member of the research team. After this process, data were analyzed using thematic analysis to identify themes that recurred across different groups. The thematic analysis was conducted as described by Braun and Clarke [17] and included the following steps: familiarization with the data, assigning preliminary codes to describe content, searching for patterns or themes across interviews, reviewing themes, defining and naming themes, and producing a report. Eight members of our research team identified initial themes. After each theme was defined, coding of all transcripts was done independently by three researchers. The three researchers that served as coders have different identity lenses with varying levels of dairy industry familiarity and experience. One of the coders considers herself a dairy industry insider as she grew up on a dairy farm, earned a Doctor in Veterinary Medicine degree, and has served the Colorado dairy industry in a variety of roles, including dairy employee training facilitator. Another coder who considers herself a dairy industry insider, did not grow up in the industry, but has a Doctoral degree in Dairy Science, with expertise in dairy calf health and behavior. While the third coder earned a Bachelor’s degree in Animal Sciences and a Master’s degree in Public Health with concentrations in epidemiology and One Health, she lacks hands-on dairy experience and considers herself a dairy industry outsider. Coding was validated with initial agreement greater than 90%; differences were discussed among coders, and agreement was reached to further refine our understanding of the concepts that emerged from the focus groups. The goal of the thematic analysis was the coding of content to identify emergent general themes that can inform future studies.

## 3. Results

Thirty-eight caretakers from five Northeastern Colorado dairy operations agreed to participate in this study. Participating dairy operations ranged in size from 750 to >5000 milking cows. Participants included dairy workers (*n* = 19), supervisors (*n* = 15), owner operators (*n* = 3), and an on-staff herd veterinarian (*n* = 1). A total of six focus groups were conducted in Spanish, with the number of participants per group ranging from three to ten. Additionally, we conducted six individual interviews with English-speaking participants. Table 1 summarizes the animal caretaker roles, number of participants, and language by focus group or interview. Focus groups averaged 39.4 min in length (range = 31.7–47.7 min), while individual interviews averaged 21.4 min in length (range = 12.5–36.4 min).

Initial review of the data identified eight recurring themes: Euthanasia Training and Occupational Safety and Health Programs, Physical Environment, Work Environment, Social Environment, Euthanasia, Animal Welfare, Human-Animal Bond, and Language Used. After coding was completed, two of the initial themes (Animal Welfare and Human-Animal Bond) were consolidated into one for a final list of seven themes. Table 2 lists the themes and definitions used for coding.

### 3.1. Euthanasia Training and Occupational Safety and Health Programs

This theme included any discussion regarding training programs, which may have included euthanasia-specific training or general occupational safety and health programs available to dairy employees, and any comments about perceived training needs. In general, training and occupational safety and health program content, delivery modality, frequency and quality varied among participating dairy operations. Additionally, perceptions in suitability of programs varied among caretaker roles within each operation.

#### 3.1.1. Euthanasia-Specific Training

Responses to the question *Is training available for the workers who perform euthanasia?* were wide-ranging. On three of the five farms, participants indicated that some form of euthanasia-specific training was available, usually from a veterinarian, and training was translated to Spanish when needed. On farms with training, workers seemed more knowledgeable of the euthanasia process and more comfortable in sharing their views.

There was a disconnect between the owner and the workers regarding the sufficiency of available training. For example, in response to *Do workers think there is enough training to feel confident performing euthanasia?*, the owner of one farm stated:

“I think there is, we have enough training to make that decision. I have no doubt about that…I’m not sure that any of my employees understand what happens other than ‘this is where you put the shot.’”

In contrast, in response to this same question, workers of the same farm answered with a simple “no.” This was a similar pattern for three of the four farms where the owners performed euthanasia; owners felt training was adequate for them but the employees did not. However, although some employees were not asked to perform euthanasia as a job-related task, they expressed a desire for euthanasia-specific training so that they could understand the decisions that resulted in euthanasia.

All but one farm indicated that they do not discuss euthanasia procedures or decisions during staff meetings. However, there was an acknowledgement by all caretakers that euthanasia should be discussed with employees more, as evidenced by the answer given by a supervisor in response to *Are there discussions about euthanasia procedures and decisions in meetings with dairy managers and co-workers?*: “we don’t talk about this [euthanasia]. I think that’s a mistake.” When asked the same question, a worker said “we don’t talk about it sufficiently…. and there is value to educating the milkers and the people” when discussing the lack of understanding in workers not involved in euthanasia procedures.

Additional comments about the availability of training in response to *Do workers think there is enough training to feel confident performing euthanasia?* highlighted the need for both reliable training materials for new employees and the possibility that workers may be asked to perform euthanasia without necessary training. For example, the owner of one farm stated, “For employees that have been here a long time, yes, but for newer employees there could be more [training]”. Additionally, a worker on another farm shared “no, but if the owner says to do it [perform euthanasia], you have to do it.”

#### 3.1.2. Occupational Safety and Health Training

Participants on all farms indicated that various types of occupational health and safety programs, not specific to euthanasia, were available. In response to *Are there worker health and safety programs at your dairy? What types of programs are there?* participants shared that safety training programs for equipment operation, animal handling, etc., were available in various formats including slide shows, on-the-job learning with either a fellow worker or manager, and FARM (Farmers Assuring Responsible Management) program materials. One participant shared, “In safety, we do our internal safety meetings about accidents that have happened, they do presentations on safety themes, safety protocols, etc. and health programs…”

However, similar to euthanasia-specific training, in two of the participating farms, there was a disconnect between manager/owner and workers regarding the availability of health and safety training. For example, a worker shared “There isn’t…from what I know, a safety course or something in case of an accident could be good, nothing more than that.” The manager of the same farm stated:

“So usually like when a new guy comes they don’t just first day hop on a tractor and take off so usually we kind of do a training program with them usually about a week long of, you know ‘here’s how you drive a tractor, here’s this’ and then we sort of do in essence, job shadowing. So, whenever we have someone new comes it’s like ‘ok so for three days you work with him and you just kind of follow him and learn what he does.’”

Workers on all farms expressed a desire for more safety training, specifically in the areas of working with cattle, general safety, first aid, and properly administering treatment to animals.

#### 3.1.3. Mental Health Resources

A lack of awareness and availability of mental health resources was evident across all farms and employee roles. For example, in response to *Are workers aware of mental health care resources available in the community?* the owner of one farm shared “No. We don’t provide that, and I would be surprised if they are aware of it. It’s just not something I’ve ever thought about.” In response to the same question, a supervisor from another farm shared:

“Honestly never, in other words as supervisors, we never touch the topic of employees, for example, ‘listen if at some time you feel depressed, problems…go to this place’ No. Never, no. Like he said, the medical insurance can cover whatever type of specialists but no we never mention it, no and I think employees in general, one never thinks to go to a psychologist.”

During a follow-up comment, potential cultural barriers to accessing mental health resources were exemplified when a fellow participant stated:

“I agree that it is something Latino and partly agriculture, that it is less common that type of…the people don’t feel…it isn’t that it doesn’t happen to us, it happens to us but we don’t feel like…we aren’t perhaps accustomed to look for that kind of help.”

### 3.2. Physical Environment

The physical environment, including extreme heat and cold, the smell of cows, and flies were identified as challenges and potential sources of stress for dairy workers of all participating farms. For example, in response to *What do workers think are some unexpected challenges that they did not anticipate when they took the job?*, workers from one farm responded with “the climate,” “being outside,” and “the degrees [temperature] where I can’t tolerate the cold.” Workers from other dairies also identified the physical environment as a source of stress: “The climate also, the sun. When it is really hot, people are very stressed…” and “…I can’t tolerate the cold and I have to tolerate it and it is a challenge for me.”

Physical environment stressors were generally recognized by managers and owners. This was exemplified when, in response to that same question, the owner of another farm stated:

“…not everyone is up to that challenge. People work in our calf area, they’re out really without much in the way of protection in the winter, in the summer. Our calf area is an open area, they, right now today it is 90 degrees. They’ll be out there working in 90 degrees. If it’s 20 below zero, they’re working in freezing and sub-freezing temperatures with water and milk and newborn calves that are wet. So those are not jobs for everybody. Working and breeding cows when its, again really really cold, nobody likes that.”

### 3.3. Work Environment

The work environment theme that emerged included discussions related to job tasks, job satisfaction, communication, and job-related stressors. Job tasks revolved around animal care and included the identification, treatment, and management of sick or injured animals. Additionally, some participants were responsible for euthanasia decisions, and most performed or assisted with the procedure itself.

#### 3.3.1. Job Satisfaction

Animal caretakers described their longevity on a specific farm as the product of good management practices. When describing their dairy experience, one supervisor stated:

“Why have I been here for 40 years? It is that they [dairy ownership] have treated me really well. Many people have asked me why I am still here. It is because they treat me well. Including now, we run well [together].”

When asked *Do workers feel their physical and mental/emotional health are valued by the dairy?*, other supervisors on the same dairy said “they are really good owners” “if you have family problems or personal ones, time [off] is given for that” and “they try to accommodate you”.

#### 3.3.2. Communication

Discussions of communication in the workplace spanned euthanasia-related tasks and tasks not related to euthanasia, as well as communication being a positive and a negative aspect of work. Regarding euthanasia, some participants expressed frustration for having to perform that specific task and perhaps wanting more effective communication. For example, one worker said “…I wouldn’t like to do it [euthanasia]. Right? But in the end what counts [is] them [management] that are the last word and they have us here to work.”

Many participants shared that communication with coworkers was valuable and contributed to a positive workplace environment. For example, in response to *Are workers generally satisfied with their jobs?*, a supervisor stated “[we] work well together because we divide work and share opinions; we share ideas and opinions when making euthanasia decisions”.

While participants saw stress as a normal part of their job, most shared that communication regarding emotional issues faced by coworkers was minimal. Some participants indicated that this kind of communication should happen between workers and upper management as needed with one supervisor saying, “I think that if they have stress, they should speak with the owners”.

One farm owner shared that one barrier to communication was gender. For example, this participant, in response to *Do supervisors check in with workers about job related stress?*, shared:

“Well for me the barrier is being a woman and being the boss. The language barrier to some point. I think that it is hard for a lot of the men, we have an older workforce now, so a lot of them are older than I am and that’s a big [problem]. And also I don’t think men are open to talking about their feelings. And especially the managers. They are managers because they are very strong and they can lead people. I think they feel like they are weak if they admit that, you know, ‘I really need some time off’ they just don’t do that.”

#### 3.3.3. Work-Related Stressors

When asked *What causes workers to feel stress related work?* lack of personnel and workload were identified as contributing factors on all participating farms. Answers from workers on one farm included “there are times where a lot of work accumulates”, “there are different tasks every day, but it is sometimes stressful” and “with a coworker that doesn’t help the team, the work starts to accumulate and then they don’t help after.”

A worker from another farm stated:

“I am [stressed] from lack of personnel and it is unorganized also. For example, we spend hours treating an animal and in the method that I do, well the sick animal gets worse, doesn’t want to eat and then you fight a lot. You stress and you have more cows to treat and everything…the majority is lack of personnel.”

As part of that same discussion, participants indicated that effective teamwork can alleviate work-related stress by saying “If there is a good work group, [people] who like to work at work and everything, the work is easy. There is no stress.”

Long work shifts, taking care of sick animals, time pressures, and poor communication with management were also identified as work-related stressors. Regarding poor communication with management, a worker from another dairy said:

“…when you see the owners upset, you are also not happy. [You] think that you work badly and [you] don’t know, we don’t even know why they are mad and they don’t give an explanation and they go to the other man, they go to the other man and the other man is then mad.”

### 3.4. Social Environment

Participants saw bringing stress home from work as a natural consequence of stress associated with being a caretaker. Referring to work-related stress, one worker said, “Well you go home with that, completely stressed all of the time”. When asked *Do workers talk to family and friends about their jobs?*, most participants identified their partners and family members as sources of support. For example, one supervisor said:

“Well you go home, and you have to de-stress with someone that is and there are times that you take [home] the bad and sometimes you reflect on it there. ‘Was that wrong? Yes, that was done wrong’. But I mean, you have to share and you have to, more than anything if you are married then with your wife.”

Some participants were concerned about sharing work-related stress with family, especially given their physical distance. For example, one dairy worker stated:

“No, if you are going to tell the family, you know [how far] we are from the family and sometimes they worry more and it causes problems with them. It is better to deal with it here, good or bad, it is for you alone, not to comment to the family.”

Another dimension of the social environment had to do with the perception that others have of the participants’ work related to on-farm euthanasia. Participants identified lack of knowledge or familiarity as reasons for differing perceptions, with one worker stating “It’s people who aren’t really familiar, they aren’t in the hospital and they don’t realize the suffering.” A coworker in agreement added, “So again, if I kill a cow, they say ‘poor thing, why did you kill it?’ It’s that they don’t know that she was suffering.”

While different perceptions from individuals foreign to the dairy industry may add some stress and the hesitation to share with others, all participants were clear on the benefits of euthanasia related to animal welfare.

### 3.5. Euthanasia

The discussion around euthanasia included protocols and procedures in place, personnel involved, the decision-making process, and preferred methods.

#### 3.5.1. Protocols, Procedures, Personnel, and Preference

Responses to *On your dairy, what types (e.g., job categories) of workers are asked to perform euthanasia?*, *Who identifies animals that need to be euthanized?*, and *Who decides about euthanizing an animal?* revealed great variation between participating farms. On one farm, the owners were solely responsible for euthanizing animals. On three farms, the owners and some supervisors euthanized cattle. On the remaining farm, an on-farm certification program allowed for supervisors and some employees to practice euthanasia. On all dairies, workers, supervisors, and owners were able to identify candidates for euthanasia, but there were varying degrees of autonomy and collaboration in making the decision to euthanize. While on some farm workers were allowed to make the decision, on some other dairies it was exclusively the responsibility of owners or supervisors. Likewise, the number of individuals assisting during the process of euthanasia varied among dairy operations. The euthanasia method used on-farm was determined by owners or supervisors and consisted of either gun shot or captive bolt gun followed by potassium chloride injection.

Workers identified their preferred methods for euthanasia as either gunshot, captive bolt gun followed by a secondary method such as potassium chloride injection or a barbiturate injection. When asked why those were their preferred euthanasia methods, workers indicated that “faster is less painful, less animal suffering”. Workers that preferred an injectable euthanasia agent spoke of previous experiences when a euthanasia with gunshot failed to kill the animal quickly (due to incorrect choice of gun and poor animal restraint) and disliking the noise produced by gunshots. Additionally, some workers mentioned that while working on other livestock operations they had observed the use of unapproved methods.

#### 3.5.2. Decision-Making

When participants were asked *Are there circumstance where workers believe euthanasia should or should not be done?* they listed indications for euthanasia which included chronic or severe disease, serious traumatic injuries, lack of response to treatment and a combination of low milk production, poor body condition, lameness and suffering that cannot be alleviated. For example, one supervisor said “when it is really sick with mastitis or pneumonia, underweight/skinny, low production, they don’t walk well and treatment was given and there was no suture; well then it is nothing more than suffering”. Another worker indicated that “[cows] are given treatment and if they don’t respond to the treatment it is when they make the decision together with the manager of euthanasia.”

When recalling a conversation with an animal welfare auditor, a worker shared “A question that they asked me once, the one from animal welfare, he said ‘if there is a cow, a cow from the milking parlor, inside fallen, broken, what would you prefer: pulling her [out of the milking parlor] or killing her now’?” The worker responded to the auditor “Kill her now.”

Participant’s hesitation to perform euthanasia because of the animal’s history or age was exemplified by this comment from a supervisor: “If it is a good cow, milker, but she is there thrown [on the ground], you don’t want…we have doubts. If you have a lot of production, your cows that fall, they slip. It hurts me to kill them.”

Making the decision to euthanize an animal on-farm was identified by a majority of the participants as the most stressful factor in the process. Participant comments included:

“The most difficult is, having to euthanize the cow because no one wants a living being to cease to exist. It is really difficult to make that decision and say ‘she has to be euthanized’. It is the most difficult for me. And the good of euthanasia is that you know that at the end the cow was going to die on its own but suffering a lot and so you make the decision to euthanize her so that she doesn’t suffer as much for as long. That is my opinion.”

“Well I think that everyone who works like that in the hospital, no one likes to make the decision to kill a cow. I think that no one, no one. But in the end we know that the animal won’t suffer anymore. I think that apart from the numbers and all of that, us hospital workers always think of that, fight so that the animal doesn’t go. And the hard decision is that: making the decision to kill her. And the benefit of euthanasia is that the animal doesn’t suffer anymore.”

Competing interests and lack of communication or established objective guidelines contributed to work-related stress and potentially resulted in animals not receiving timely euthanasia. For example, a supervisor stated:

“… [do] I sell her tomorrow for meat or I kill her today, I euthanize today? That is the decision that is also very difficult because you are like ‘do I wait until tomorrow or is she going to suffer overnight?’.”

A worker added to the discussion and highlighted the potential effects on timely euthanasia when a euthanasia decision tree with clear end points is not available “If the decision is not clear, we give them another day.”

While euthanasia can be a source of stress and an unpleasant experience for dairy caretakers, participants understand its importance with one worker summarizing this conflict by stating “First of all, this is not a pretty job. Euthanasia. Nobody wants to do it, but it has to happen.”

### 3.6. Animal Welfare and Human-Animal Bond

Human–animal bonds were evident in all participants regardless of training or experience. Workers clearly demonstrated empathy and compared cattle with humans and pets in the suffering experienced with disease or traumatic injuries and the relief that death can bring in certain situations.

Caretakers spoke of animals deserving kindness and euthanasia being a way to practice that kindness. For example, one worker stated: “They are the same as animals, dogs and cats. If they are sick, we take them to the veterinarian and they give them medicine and they get put to sleep.” Another worker explained:

“Like my partner says the decision is the hardest, the most difficult. It is for the good of the cow. Here if we make the decision to kill a cow it is because the cow really isn’t going anymore like we say here. Even if you want to sell, she won’t go. If we are seeing that she is…that she won’t get up, we make the decision, and it is for her own good.”

And while discussing reasons for euthanasia, a worker simply added, “And she is feeling pain.”

Regardless of size of dairy operation, dairy caretakers recognized that fondness for some animals affected their emotions around euthanasia. For example, one supervisor shared:

“You become fond of them. Yes. You become fond of an animal. It hurts me. It is normal. It’s that when you become fond of someone and they go down, well it is going to hurt. They are feelings, all of us have feelings. It is normal. But the decision is so that they don’t suffer.”

Two other supervisors contributed to the discussion by saying, “There are cows that are really lovable. You remember the number on the ear tags of that cow.” and “There are some cows that are friends and you arrive and they greet you. When they cease to exist, they are missed.”

### 3.7. Language Used

Terms and phrases used and the comfort level while describing the act of euthanasia and the various methods and tools required varied among dairy operations. While caretakers routinely employ euphemisms such as “put to sleep” or “put them down”, caretakers on operations with more formalized euthanasia training tend to use more technical and accurate language. When discussing preferred methods, workers with more training referred to “captive bolt gun”, “potassium chloride” and barbiturates specifically. On the other hand, workers without formal training referred to “injection” or “medicine” while describing injectable euthanasia agents, and “gun” or “spear” while describing physical euthanasia agents such as a penetrating captive bolt gun but did not provide specific descriptions of chemicals or the different firearms used for euthanasia of cattle.

## 4. Discussion

While by design, the information derived from this qualitative pilot study is not generalizable and doesn’t allow for quantifiable differences between farms, it is rich and provides depth necessary to inform future studies. Moreover, study results support our hypotheses that euthanasia training and management (1) vary among the participating dairy operations and (2) are correlated with caretaker knowledge, attitudes and perceptions regarding euthanasia.

Euthanasia training for dairy workers was varied and sometimes lacked in content, format, and quality. This finding is congruent with previous research on dairy worker training in general [12,13,18] and on the need for euthanasia-related training [19,20]. Current management practices too often result in delayed euthanasia or unassisted death for significant number of dairy animals with conditions that carry poor prognosis and warrant euthanasia (e.g., nonambulatory cows) [21]. It is important that caretakers not only understand how to perform euthanasia but also ‘why’ it is critical to follow euthanasia procedures [22]; this can improve comfort level of workers performing the task [23], prepare them for what they will observe [24], and help them understand the value of their work [22]. On dairies where workers haven’t been properly trained because only the owner or the owner and a selected group of employees are responsible for euthanasia, communication and timely euthanasia decisions could be severely hindered [9]. Besides the negative impact to animal welfare, limited euthanasia-related training and communication could contribute to distress in those workers who don’t understand the procedure.

Workers expressed an interest in learning more about euthanasia and management recognized limited training and communication as problematic. Dairy caretakers who lacked training tended to use euphemisms and were unsure of specifics of the euthanasia process. Euphemisms, such as “putting an animal to sleep” or “sacrificing an animal,” are used to lessen the emotional response that accompanies making decisions about and/or performing euthanasia [25]. Our findings are consistent with previous studies that demonstrate animal caretaker interest for more euthanasia-related training in other livestock industries [23,26]. Moreover, while veterinarians are seldomly consulted or involved in euthanasia decision-making and training on dairy operations [20], recent survey data suggests that veterinarians want to be included in the creation and facilitation of training programs [27,28]. Involvement of veterinarians could help workers feel more comfortable with technical language and more knowledgeable about the importance of timely euthanasia, indications for the procedure and approved methods to humanely stop animal suffering. Although veterinarians were eligible to participate in our study, only one who served as on-staff veterinarian for one of the participating dairies did. The other eligible veterinarians were not on farm during data collection. While limited time spent on farm can pose a challenge to engagement in euthanasia training and discussions, veterinarian input could prove extremely valuable. Future studies should explore the barriers associated with limited veterinarian involvement and identify strategies for more effective partnerships between farm personnel and veterinarians.

Earlier work has highlighted best practices for dairy worker engagement and training [29] and the effects on worker perceptions, knowledge, and behavior when the training interventions are designed with cultural congruence in mind and with the input of workers [30]. Culturally congruent trainings should extend beyond euthanasia and include broader occupational safety and health topics. Our data highlighted discrepancies between worker and management perceptions within a dairy operation on the availability and suitability of occupational safety and health training and support programs. More effective programming and communication should help decrease knowledge gaps and improve caretaker’s occupational safety and health.

In addition to the need for more consistent euthanasia-related and occupational safety and health training for all animal caretakers, our data clearly shows necessity for more effective communication regarding euthanasia decisions and how those relate to caretaker safety and well-being. Providing a space for honest and constructive discussion could help reduce conflicts and feelings of distress and failure among dairy caretakers. One of the challenges with euthanasia that has been identified by animal caretakers is the emotional burden of ending an animal’s life even when knowing that euthanasia is the best option to end animal suffering [3,5,27,31,32,33]. As seen in participant responses, this emotional burden is exacerbated by co-workers who are not familiar with or do not understand decision-making related to euthanasia. In a study with shelter workers, participants identified that support (i.e., “more understanding and less criticism”) from individuals who did not have euthanasia responsibilities would be beneficial to employee well-being [15]. Additionally, Reeve et al. [34] relates the social stigma of performing “dirty work” [35] to animal shelter workers who must euthanize animals as part of their job and this concept has similarities to the social dimension described by participants in the current study. Furthermore, the lack of awareness of available mental health resources coupled with the limited discussions with coworkers and management, and the strains that sharing with family might put on personal relationships and social networks stress the need for on-farm interventions. While the emotional burden of euthanasia has been explored in other settings, especially in veterinary practices and animal shelters [2,3,4,5], on-farm interventions that could alleviate work-related stressors and promote greater emotional well-being, positive personal relationships and greater productivity are needed.

On-farm interventions should not only encourage animal caretakers to share feelings about euthanasia and other work-related tasks, but also be a space for farm employees to discuss other challenges related to the physical farm environment, teamwork, communication, and job organization. All these topics emerged as challenges and stressors in this study as well as previous ones [12] and should be considered in an effort to improved occupational safety and health and overall employee satisfaction. On-farm interventions to alleviate work-related stress could include peer discussion sessions where caretakers share successes, failures, and discuss concerns, as well as regular staff meetings where mental health resources available on-farm or in the community are discussed with all employees. While participants in this study indicated that cultural nuances might prevent dairy employees from seeking mental health care, the normalization of this topic could prompt animal caretakers to access much needed resources.

While we enjoyed a positive working relationship with the participating farms and created an environment conducive to honest sharing that resulted in rich information, data collection sessions concluded in a relatively short amount of time and it is possible that caretakers’ level of messaging was limited. Although dairy ownership was supportive of caretaker participation and allowed for focus groups and interviews to occur during normal work shifts, caretakers could have shortened their answers in an effort to return to their daily routines. Dairy workers have previously identified a strong sense of responsibility and pressures related to diverse job tasks [12] which may have limited caretaker participation in this study. The group dynamics in any focus group could also affect participation. While we encouraged all participants to share, in some instances they would just state agreement with previous comments. Additionally, the fact that caretakers in four of the five dairy operations had never discussed euthanasia or their feelings about it in a group setting could have affected the substance of the caretakers’ contributions to the focus group sessions. More opportunities to share perceptions about euthanasia and other work-related tasks could serve both the quality of the data captured in future studies as well as the performance and well-being of dairy caretakers.

Additional strategies that could decrease the stressors specifically related to the euthanasia decision-making process include culturally responsive training interventions, printed resources such as decision trees or flow charts posted in common areas of the dairy, enhanced record systems that allow for more objective euthanasia decisions, regular meetings with management to discuss difficult cases and jointly decide on next steps. Given the clear human-animal bond and empathy expressed by all participants, the goal of interventions and support systems should be to minimize the burden of the decision-making for the caretaker to alleviate conflict and feelings of guilt or failure.

Future research should aim to test and validate potential on-farm interventions for their effectiveness in reducing euthanasia-related stress, improving the euthanasia decision-making and practice, and increasing caretaker and animal wellbeing on dairy operations. Furthermore, future work should evaluate how culturally responsive on-farm interventions might affect dairy employee perceptions about mental health resources as a tool to improve their wellbeing.

## 5. Conclusions

This pilot study yielded valuable in-depth information to be used in future studies. All focus group and interview participants demonstrated a human–animal bond and sense of empathy for dairy animals and a clear understanding of euthanasia as an important tool to humanely stop animal suffering. Training was associated with knowledge of euthanasia and the language used to describe the process. Our data clearly highlighted the need for culturally responsive interventions to address training needs, euthanasia-related stressors, and the lack of mental health resources awareness of dairy caretakers. Future studies should target the confirmation of our findings across a larger population of dairy caretakers. Research aimed at validating the efficacy of interventions for this community of animal caretakers should offer much needed strategies to improve human and animal wellbeing on dairy operations.

## Figures and Tables

**Table 1:** Participant role, number, and language by focus group or interview session.

Session	Role	Number	Language
Focus group 1	Supervisors	5	Spanish
Interview 1	Owner operator	1	English
Focus group 2	Supervisors	5	Spanish
Interview 2	Owner operator	1	English
Focus group 3	Dairy workers	5	Spanish
Interview 3	Owner operator	1	English
Focus group 4	Dairy workers	3	Spanish
Interview 4	Dairy worker	1	English
Focus group 5	Supervisors	4	Spanish
Interview 5	Supervisor	1	English
Focus group 6	Dairy workers	10	Spanish
Interview 6	Veterinarian	1	English

**Table 2:** Themes and definitions used for coding of focus groups and interview transcripts.

Theme	Definition
Euthanasia Training and Occupational Safety and Health Programs	Comments about euthanasia training and occupational safety and health (OSH) programs available to caretakers as well as perceived training needs
Physical Environment	Comments related to climate (weather), water, soil, and air
Work Environment	Comments related to job tasks, job satisfaction, communication, and job related stressors
Social Environment	Comments about support provided by family and friends, public perception regarding their job tasks/euthanasia and communication with outside stakeholders
Euthanasia	Comments regarding euthanasia protocols and procedures, decision-making process, and caretakers responsible for performing euthanasia
Animal Welfare and Human-Animal Bond	Comments revealing empathy towards animals and a close human-animal bond, and detailing caretaker perceptions on the process of euthanasia
Language Used	Terms and phrases that caretakers utilize to describe the process of euthanasia

## Data Availability

The data presented in this study are available on request from the corresponding author. The data are not publicly available due to confidentiality restrictions.

## Appendix A

1. Focus Groups/Interview Questions: On your dairy, what types (e.g., job categories) of workers are asked to perform euthanasia?
2. How often are these workers asked to perform or help someone perform euthanasia?
3. Who identifies animals that need to be euthanized?
4. Who decides about euthanizing an animal?
5. How is the method for euthanasia determined?
6. Are there methods that are preferred by the workers who perform euthanasia?
7. Is training available for the workers who perform euthanasia?
8. Do you know what languages are used in the trainings?
9. Are there discussions about euthanasia procedures and decisions in meetings with dairy managers and co-workers?
10. Do workers think there is enough training to feel confident performing euthanasia?
11. What causes workers to feel stress related to work?
12. Are there worker health and safety programs at your dairy? What types of programs are there?
13. Do supervisors check in with workers about job related stress?
14. Do workers feel physically safe at work?
15. Do workers feel their physical and mental/emotional health are valued by the dairy?
16. Are workers generally satisfied with their jobs?
17. What do workers think are some unexpected challenges that they did not anticipate when they took the job?
18. Do workers talk to family and friends about their jobs?
19. Are workers aware of mental health care resources available in the community?
20. In general, what are workers thoughts about euthanasia, thinking about the animals and each other?

- Are there aspects of the process that cause workers distress or that they would not want to do?
- Do workers think there are positive aspects of euthanasia?
  Are there circumstances where workers believe euthanasia should or should not be done?
